# Feasibility of cognitive testing and ecological momentary assessments using smartphones in middle aged and older adults with insomnia

**DOI:** 10.1186/s44247-025-00158-4

**Published:** 2025-07-01

**Authors:** Sara Ghadimi, Jason Ereso, Alexander J. Kaula, Nick Taptiklis, Francesca Cormack, Cathy Alessi, Jennifer L. Martin, Joseph M. Dzierzewski, Arash Naeim, Sarah Kremen, Tue Te, Constance H. Fung

**Affiliations:** 1 University of California, Los Angeles, CA USA; 2 VA Greater Los Angeles Healthcare System, Los Angeles, CA USA; 3 Cambridge Cognition, Cambridge, UK; 4 National Sleep Foundation, Washington, District of Columbia, USA; 5 Cedars-Sinai Medical Center, Los Angeles, CA USA

**Keywords:** Cognitive assessment, Insomnia, Hypnotics, Mobile devices

## Abstract

**Background:**

Older adults with insomnia who use benzodiazepine receptor agonists (BZAs) may be at increased risk of cognitive impairment. Cognitive testing outside of clinical settings may yield results that are more reflective of individuals’ cognition in their natural environment, where they experience fluctuations in mental state (e.g. drowsiness). We assessed the feasibility of self-administered cognitive testing via smartphone apps for collecting in-moment, in-context data about a person’s current state (ecological momentary assessment, EMA).

**Methods:**

Participants (*n* = 20; median age 66 years; 14 females, 18 white) aged ≥ 55 years who were recruited from a BZA deprescribing trial were invited to complete (over a 28 day period) daily drowsiness assessments on an EMA app (cued by smartwatch alerts) and weekly self-administered digit span (DGS) forward/backward (2 [minimum] – 9 [maximum]), verbal paired associates (VPA; 0 [best]—24 [worst] total errors), and cued delayed recall of VPA (VPA-DR; 0 [best] – 8 [worst] errors) tests on a cognitive app. We assessed the completion of EMA (0–28 days) and cognitive sessions (# of participants per # sessions completed). We performed thematic analysis of the participant interviews.

**Results:**

The median number of days that EMA was completed was 24.5. Twelve (60%) individuals participated in 4 sessions; 2 (10%) individuals participated in 3 sessions; 2 (10%) individuals participated in 2 sessions; and 4 (20%) individuals participated in 1 session. No drowsiness was reported 36% of the time, whereas 38% of the responses reflected feeling “a little bit” drowsy and 26% at least “somewhat” drowsy. Mean cognitive test scores were DGS-Forward = 7 (SD 1.3), DGS-Backward = 5.6 (SD 1.0), VPA total errors = 9.9 (SD 3.7), and VPA-DR = 2.2 (SD 1.9). Three themes emerged from the participant interviews: 1) concern for one’s own cognitive abilities, 2) strategies employed for optimizing scores (including strategies that would invalidate results), and 3) ease of use of the applications.

**Conclusions:**

Our findings indicate that mobile cognitive tests and EMAs are feasible in this older population. Further work is needed to understand how scores are influenced by the setting, mood, and behaviors.

**Supplementary Information:**

The online version contains supplementary material available at 10.1186/s44247-025-00158-4.

## Background

Although in-person, structured, pencil-and-paper cognitive testing is the gold standard approach for cognitive assessment, interest in computerized cognitive testing (e.g., smartphone, tablet, desktop platforms) has been growing. Computerized cognitive testing provides the opportunity to collect conventional cognitive measures in new and improved ways (e.g., algorithms to allow for adaptive tests, automatic scoring, scalable in different languages) to allow for more widescale, longer periods of follow up [1].

Mobile computerized cognitive testing outside of a clinical or research setting in individuals’ natural environments has the added advantage of assessing their cognition in a more person-centric manner—the location and environment where they carry out their daily activities. Inferences from testing in these environments have the potential to predict how well individuals function in their daily lives [2]. Mobile cognitive testing can be readily paired with data from “wearable” devices, which are increasingly used in middle-aged and older adult populations, and facilitate repeated sampling of behavior and experiences in real time—ecological momentary assessments (EMA). EMA administered concurrently with mobile cognitive testing offers the opportunity to strategically sample and explore multiple times the real world effects of current experiences, symptoms, physiological states and behaviors in real-time [3]. A recent systematic review of mobile cognitive assessments found that these EMAs could yield novel and important information [4].

Studies of self-administered mobile cognitive assessments in clinical and non-clinical populations are promising, with an overall adherence rate of 79%, high levels of between- and within-person reliability and high levels of convergent and discriminant validity with in-person assessments [4]. Although the feasibility of using mobile technology to measure cognition has been assessed [5–7], to our knowledge, feasibility studies of mobile cognitive assessment and EMA have not been conducted in middle-aged and older adults with insomnia and chronic benzodiazepine receptor agonist (BZA) medication use. This population is at increased risk of cognitive impairment, and variable use of hypnotics and fluctuations in sleep disturbances have the potential to cause daytime somnolence [8] and fluctuations in cognitive performance [9]. We aim to: 1) assess the feasibility of administering cognitive testing and EMA in this population, 2) determine whether individuals would complete the testing/assessments, and 3) understand their unique needs and perspectives.

## Methods

### Participants and setting

Participants were recruited from a clinical trial that used 8 weekly sessions of cognitive behavioral therapy for insomnia (CBT-I) to help adults ≥ 55 years old sleep better and reduce their use of BZAs, which was conducted at the University of California, Los Angeles (clinicaltrials.gov NCT03687086, registered September 27, 2018). Participants provided separate written informed consent for the feasibility study. Participants of the current study were community-dwelling adults with a Mini-Mental State Examination (MMSE) score in the normal range (24–30). This MMSE threshold was used in the parent study to exclude participants who might be poor candidates for CBT-I and a new method of medication tapering that involved use of a blinded/masked taper [10]. The Pittsburgh Sleep Quality Index (PSQI) [11, 12] was administered as an assessment for the clinical trial. All study procedures for the current feasibility study were approved by the University of California, Los Angeles institutional review board (IRB#20–001519).

### EMA procedures

After providing written informed consent, participants were given a study iPhone SE (Apple, Cupertino, California, USA) and Apple Watch 5 or 6 (Apple, Cupertino, California, USA) or used their own iPhone and Apple Watch for the study. Participants had the option of using print-out instructions to set up the devices if they were mailed to them, or they could set up an appointment with a research staff member to go over the steps over a video or phone call. For 28 days at a time pre-determined by the participant, a notification on the Apple Watch prompted them to complete daily EMA questions at that moment through the Status/Post app (Infinite Arms, Charleston, South Carolina, USA), which was pre-loaded on study-owned iPhones, or downloaded by participants onto their personal mobile device. We included EMA questions that have been published as well as de novo questions. EMA questions assessed their current location, level of drowsiness in the past 2 h [13], current thinking abilities, current mood [13], and current level of fatigue [13], number of alcohol and caffeinated beverages consumed that day, and whether they used any tobacco products that day. The cadence of the EMA surveys and survey data were managed and collected through Research Electronic Data Capture (REDCap) electronic data capture tools hosted at the University of California, Los Angeles and stored on a university research server. The Status/Post app integrates REDCap with Apple’s iOS.

### Cognitive testing procedures

**Fig. 1:**
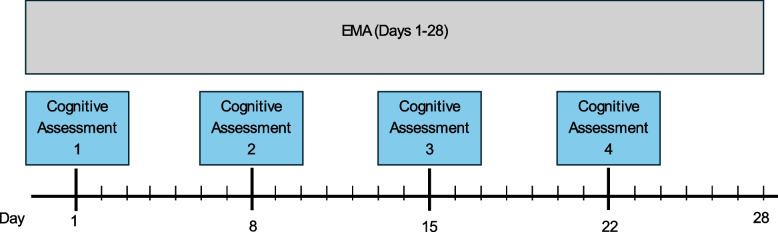
Provides a timeline of study activities (ecological momentary assessments [EMA] and self-administered cognitive assessments)

Once a week (e.g., days 1, 8, 15, 22) participants were directed to complete cognitive assessments and were asked if they are wearing headphones/headset/earbuds (in addition to the usual EMA questions). Figure 1 shows the timeline of EMA and cognitive testing activities for participants.

The actual date the cognitive assessment was completed was logged automatically. Although participants were only invited to participate in a total of 4 cognitive testing sessions (once per week), some participants opted to and successfully completed them more frequently (one participant completed 9 sessions and three participants completed 5 sessions) due to a software error. Participants who completed at least 4 cognitive tests have been grouped together in the analyses. Cognitive assessment was completed through a unique link to the Neurovocalix website (Cambridge Cognition, Cambridge, United Kingdom) that was pinned to the participants’ device home screen to simulate an app. Participants’ working and episodic memory was assessed through recorded verbal responses to Digit Span Forward (DGS-F) and Backward (DGS-B) tests, which entails providing participants with a random series of digits auditorily and asking the participant to repeat the digits in the order presented (forward span tests working memory and attention) or in reverse order (backwards span tests working memory, attention, cognitive control, and executive function). The longest sequence correctly achieved is measured (2 [minimum] – 9 [maximum]). Assessment of associative and episodic memory was measured by tallying total and delayed recall errors on the Verbal Paired Associates (VPA) test. This task presents participants with a set of eight word-pairs auditorily and then asks the participant to remember which target word was paired with each prompt word. If they do not correctly recall all eight pairs, they are presented with the pairs two additional times. In the delayed recall phase (VPA-DR), they are asked to recall the learned word pairs without hearing the list again. The number of errors during each attempt is counted as well as the number of attempts, yielding a VPA total error score (totaled for the three attempts) ranging from 0 (best) to 24 (worst) total errors and VPA-DR score ranging from 0 (best) to 8 (worst) errors.

### Interviews

At the end of the 28-day period, participants were invited to complete a voice-recorded semi-structure interview to receive feedback on their experiences. An interview guide that was adapted from a published guide was used (see Supplement Interview Guide) [14]. Two research team members (SG, JE) performed thematic analysis of the interview audio recordings under the guidance of the lead investigator (CF).

### Statistical analyses

Statistical analyses were conducted by the university-based research team (Python 3.10, NumPy, Pandas, Seaborn, Scipy, Random, Requests, IPython packages). Descriptive statistics (mean, standard deviation (SD), range, frequency) for demographic variables were calculated and the number of participants who underwent testing was summarized. Mean DGS-F and DGS-B scores (2 [worst] to 9 [best]), VPA total errors over 3 attempts (0 [best] to 24 [worst]), and VPA delayed recall were calculated. Test–retest reliability was calculated (Spearman’s *r*), comparing the test results for session 1 (s1 on day 1) with test results for sessions 2 (s2 on day 8), 3 (s3 on day 15), and 4 (s4 on day 22). A non-parametric measure of correlation, Spearman’s *r*, was selected because of the modest sample size and appropriateness for data that may be skewed or have non-linear relationships.

## Results

Of the 20 enrolled participants (mean age 67 SD 6.7 years, median age 66 years, range 56–85 years; mean and median education 17 years; 14 females; 18 white), 12 participants underwent at least 4 cognitive assessments, 2 underwent 3 assessments, 2 underwent 2 assessments, and 4 underwent only 1 assessment (Table 1).

**Table 1:** Adherence to cognitive assessments

Number of cognitive test sessions	Number of participants	Percentage of participants
4^a^	12	60%
3	2	10%
2	2	10%
1	4	20%

^a^Participated in at least 4 cognitive test sessions (some participants were able to participate in more than 4 sessions due to an unexpected software issue)

Of the 12 participants who underwent at least 4 cognitive assessments, 5 participants performed the assessments in exactly one-week intervals as instructed (the remaining performed the assessments either 1 day early or 2 to 5 days late). Mean PSQI global score was 11.3 (SD 4.2; min 3, max 18; *n* = 20), with PSQI sleep efficiency factor mean 2.7 (SD 1.9, min 0, max 6), perceived sleep quality factor mean 6.5 (SD 2.0, min 3, max 9), and daily disturbances mean 2.2 (SD 1.0, min 0, max 4).

**Fig. 2:**
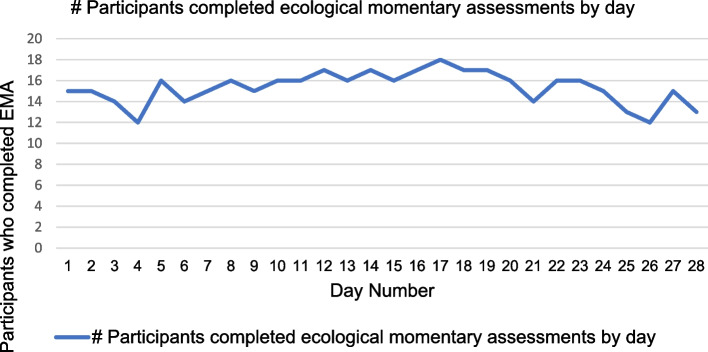
Shows adherence data for ecological momentary assessments analyzed per day (max = 18 participants on day 17, min = 12 participants on days

The median number of EMA completed per participant was 24.5 (mean 21.5; SD = 7.3; interquartile range 6.75; mode 25; maximum possible = 28). The EMA adherence data per day are displayed in Fig. 2.

Three participants completed all 28 days of EMA as instructed. The most common location participants reported completing EMA was at a home, either their own or of someone they knew (81.4% of total EMA). When asked about drowsiness, 36.4% of the time, participants reported their drowsiness level as “none”, 38% of the time as “a little bit”, and 25.6% of the time as at least “somewhat.” Nearly half (46.2%) of responses reflected participants being a little bit fatigued, 28.4% not at all fatigued, and 25.4% at least somewhat fatigued. Participants reported their current thinking ability to be good or very good 83.4% of the time. 84.8% of responses indicated that the participants’ mood was good or very good.

**Fig. 3:**
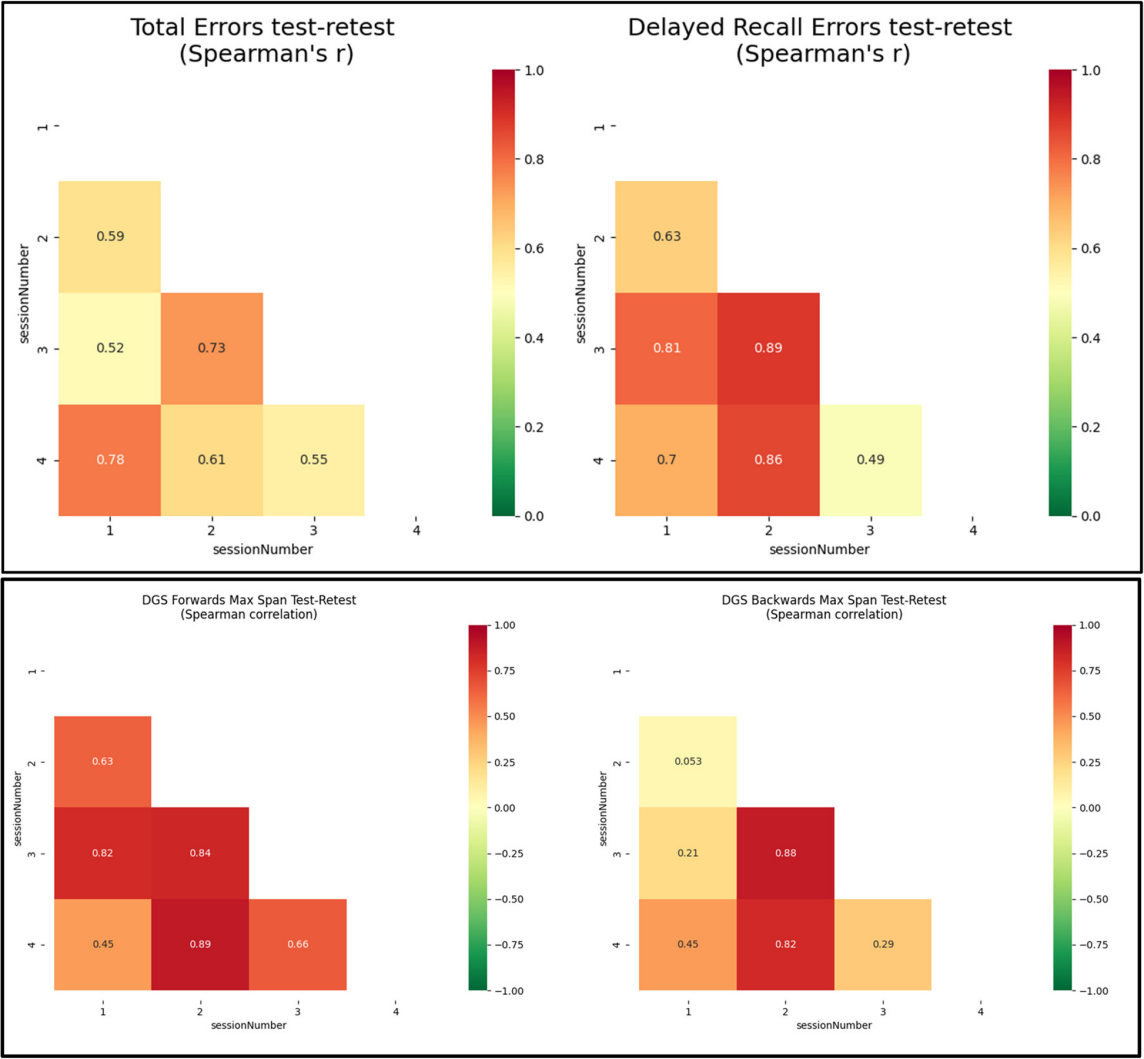
Shows the test–retest reliability for Digit Span Forwards, Digit Span Backwards, Verbal Paired Associates Total Errors (over three attempts), and Verbal Paired Associated Delayed recall Errors from session 1 to session 4

Mean scores were DGS-F = 7 (SD 1.3), DGS-B = 5.6 (SD 1.0), VPA total errors = 9.9 (SD 3.7), VPA delayed recall errors = 2.2 (SD 1.9). Test–retest reliability (*r*) was 0.63 (s1,2), 0.82 (s1,3), and 0.45 (s1,4) for DGS-F and 0.05 (s1,2), 0.21 (s1,3), 0.45 (s1,4) for DGS-B. Test–retest reliability for VPA total errors were 0.59 (s1,2), 0.52 (s1,3), 0.78 (s1,4) and for delayed recall VPA errors were > 0.63 for s1 vs s2-s4 (Fig. 3).

Three themes emerged from the participant interviews (Table 2): 1) concern for cognitive abilities, 2) strategies employed for optimizing scores and response to experience, and 3) ease of use of the applications.

**Table 2:** Participant experience with mobile cognitive testing and ecological momentary assessment: Themes from semi-structured interviews

Theme 1: Concern for cognitive abilities	
Quote	Tie-in
“I’m a little annoyed because I think I’m…smart…, right? Then I start going through this and I’m going, ‘God, if someone looked at this they’re thinking I’m a moron!’ So I don’t like this to start with because it’s making me feel bad”	Due to the difficulty of the cognitive assessment, this participant became concerned that others might think differently of their intelligence if they saw their results
“You worry that there is something wrong with you”	This participant started worrying about their own cognitive abilities due to the difficulty of the cognitive assessment
Theme 2: Strategies employed and response to experience	
Quote	Tie-in
“Yeah, I did cheat. I tried not to but I got so frustrated at the end”	The participant expressed a level of high frustration with the difficulty of the cognitive assessment and in response, wrote down the answers so they could perform better
“The first time it was a little difficult, but then the second time I knew what to expect and it was more comfortable”	This participant initially did not know what to expect from the cognitive test. Once they became more familiar with the procedures, the test was not as difficult for them
Theme 3: Ease of use of applications	
Quote	Tie-in
“I was very- in need of a lot of assistance a lot of time and every time you really helped me”	The participant liked being able to reliably contact the study team with technical support questions in order to complete study activities
“It would be nice to have after hours [technical support]. That would be I think helpful for people that you know, they need to talk to somebody”	Technical support after working hours might be needed to address participants’ concerns with their technology

For theme 1, eight participants spoke about some level of concern for their own cognitive abilities after engaging with the cognitive assessments. Related to theme 2, seven participants (35%) admitted that they wrote down the items they were asked to remember (i.e., cheated) or they at least thought about writing down the items to improve their test performance. Eight participants (40%) said they adjusted their strategy or approach to answering the questions in some way to do better. Five participants (25%) wanted more tests than the four sessions they were given (for example, to improve their scores with practice). Regarding the third theme, seven participants (35%) said they needed extra technical help from the research team at some point during the study. Three participants (15%) said the enunciation or volume of the digital voice that was administering the assessment was poor. Ten participants (50%) said the pre-determined time of day that the surveys and assessments were scheduled was important.

## Discussion

To our knowledge, this is the first study that evaluated the feasibility of self-administered mobile cognitive tests and EMAs in middle-aged and older adults with insomnia. All participants completed at least one cognitive test session and 60% completed all 4 cognitive sessions. The median number of EMAs completed was 24.5 days (88% days). The EMA assessments provide insight into the mental and physical state of the individuals during testing—over one-third (38%) of the participants reported that they were drowsy and 46% of the participants reported being fatigued. Our findings also provide pilot data on the environment selected by participants for completing testing. A majority (81%) of the EMAs were completed at home and half of the participants reported that the timing of the tests mattered. Our findings indicate that remote cognitive testing and EMA assessments, while feasible, could be further optimized by addressing several barriers.

The interviews with participants provided insight into possible barriers to assessing cognition in a natural environment using mobile apps in this population. These include the potential for individuals to have negative emotions (e.g., anxiety about test performance or concern about memory impairment) during assessments, leading to use of strategies that may invalidate the test results (e.g., making written notes) or quitting the test before completion. These are important factors to be aware of when implementing digital health assessments for older adults as they can affect accuracy of results. A study by Moore of older adults undergoing mobile cognitive assessments found six events where invalid strategies were used or suspected (in their study, participants were suspected of receiving assistance from non-participants), although these events represented less than 1% of events [15]. The negative emotions could explain why 40% of participants did not complete all 4 sessions—these negative emotions may have contributed directly to nonadherence by reducing motivation to complete additional sessions or indirectly, by reducing motivation to seek assistance for overcoming other challenges such as software issues (e.g., accessing the app). Participants’ responses to testing, however, varied. Interestingly, the interview results revealed that 8 participants adjusted their strategy when performing the cognitive tests and five participants reported that they wanted to complete more than four sessions of cognitive tests (e.g., to improve their memory). Anticipatory guidance in a future study that prepares participants to handle negative emotions would be one strategy to increase adherence to the testing program. The level of EMA daily completion was relatively high and may reflect the relative ease and neutral emotions associated with completing EMAs.

With the increasing use of telehealth programs, it is important to reduce barriers for patients who have time, mobility, and distance constraints. The high rates of completing at least one cognitive session suggest that this method of testing cognition outside of the clinic is promising. Pairing EMAs with mobile cognitive tests can help build an understanding of how behaviors and conditions such as insomnia influence cognition. Our study also demonstrated that cognitive tests and EMAs can be performed independently as more than half of the participants did not report technological difficulties. A previous study using remote cognitive tests and EMAs required participants to complete visits through video, which requires staff to be onsite and monitor each participant [16]. The approach tested in the current study could help reduce the cost of care and reduce staffing needs.

The test–retest reliability for DGS-B was in the low range and was noted to be much lower than that of the other cognitive tests (DGS-F, VPA, and delayed recall VPA). DGS-B requires more complex processing than DGS-F. Individuals who are distracted by stimuli in their environment, which could vary across assessments, may be more likely to have fluctuating scores. Some individuals use visual rather than verbal cognitive strategies for the DGS-B [17], and sleep time, which can fluctuate, could impact visuospatial processes [18]. Heightened anxiety during the more complex DGS-B task could also decrease performance [19]. Finally, a more difficult task might lead to increased reliance on unapproved strategies (e.g., writing down the digits), but use of unapproved strategies could also vary across assessments based on availability of a writing instrument. We speculate that the low test–retest reliability observed in our study could result from one of these explanations; however, given the modest sample size of the current study, the issue deserves further investigation and replication.

Several challenges and limitations were identified in the study. Although not a primary goal of the study, we were interested in the accuracy of the self-administered mobile cognitive tests for this population, but our procedures did not include concurrent in-person pen-and-paper testing that would allow for comparisons. Future studies should include procedures that would enable the comparison of an in-person cognitive test with mobile cognitive test results to determine if there are significant differences between the two settings and tests. An unexpected finding from the interviews was that seven participants admitted to using strategies that would invalidate their scores (e.g., writing down the word pairs or number sequences). Algorithms to detect the use of strategies that would invalidate test results are needed if testing is unsupervised, or alternatively, mobile testing via smartphones could include a video recording of the session. In addition, the results from the post-interview indicated that seven participants reported needing technical help while volunteering for the study. Technical issues near the end of the project prevented 3 participants from accessing the cognitive testing app, which may have influenced the number of participants completing cognitive tests. A dedicated staff member available to support participants experiencing technical issues would be crucial for future programs. Given that three participants also reported that they had issues with hearing the responses from the device, a feature that allows participants to have greater control over the volume would ensure that the sound volume is not influencing scores or the participant’s experience with the app. Adding a few items that test the participant’s ability to hear the stimuli or to provide feedback about difficulty hearing stimuli could be helpful for interpreting the results. Adding a recognition task after the delayed recall portion of the VPA could provide information about whether memory impairment is due to retrieval only (factors such as medication, somnolence, and cerebrovascular disease may be more likely to cause recall errors) or encoding. Expanding the number of EMA assessments to up to four times per day as described in a recent publication would capture more variation across the day [13]. Finally, future studies should recruit participants from different ethnicities, genders, and education levels to expand the generalizability of the findings.

## Conclusion

Our study assessed the feasibility of testing cognition outside of the clinic and collecting EMA data in older adults with insomnia using mobile devices over a four-week period. Although most participants were able to complete at least one cognitive testing session and provide EMA data on most days, several barriers including concern about their cognition and technical issues limited participants from consistently completing testing and EMAs. Future work is needed to understand how to address the barriers identified in our study and to explore how cognitive performance in an individual’s natural setting is influenced by the setting, physical and mental state, behaviors and other factors.

## Supplementary Information

Supplementary Material 1.

## Data Availability

The datasets used and/or analysed during the current study are available from the corresponding author on reasonable request.

## Abbreviations

BZA: Benzodiazepine receptor agonists
CBT-I: Cognitive behavioral therapy for insomnia
DGS-F: Digit span forward
DGS-B: Digit span backward
EMA: Ecological momentary assessments
MMSE: Mini-mental state examination
PSQI: Pittsburgh sleep quality index
REDCap: Research electronic data capture
SD: Standard deviation
VPA: Verbal paired associates
