# Supplementary material for: Feasibility of cognitive testing and ecological momentary assessments using smartphones in middle aged and older adults with insomnia
Source: BMC Digit Health. 2025 Jul 1;3(1):21. doi: 10.1186/s44247-025-00158-4 (PMC12208955; doi:10.1186/s44247-025-00158-4)
Supplement: Supplementary file 1 — Supplementary Material 1. [file 44247_2025_158_MOESM1_ESM.docx]

**BRAIN-e semi-structured interview guide**

1. Introduction:
   1. Introduce research staff conducting interview
   2. Reiterate volunteer nature of interview
   3. Ask for permission to record interview: __Yes ____No
   4. Describe expected length of interview
   5. Provide brief outline of interview session
2. Topics

   Participant initials:

Date of interview:

Staff present:

Recorded (y/n):

- 1. What is your prior experience using:
     1. iPhone, other smartphone, Apple Watch, other smartwatch
     2. Any digital brain health/cognitive testing
  2. What was your experience receiving the devices from the research team and getting set up to do the brain health tests?
     1. Prompt: Instructions, packaging, communication with team
  3. What was your experience using the cognitive testing apps/website, Apple Watch
     1. Prompt: enjoyable? Interfered with daily tasks?
  4. What was challenging
     1. Software/user interface: How were the instructions, what was it like navigating the software?
        1. Prompt: Any issues with using fingers/hands to respond to questions, any hearing issues, any problems seeing the problems?
        2. Prompt: Any changes in your approach over the course of the month?
     2. Content: Describe your level of interest in the tests?
        1. Prompt: boring, game-like
  5. If you encountered problems, what did you do? What type of support did you seek? What was the outcome?
  6. Suggestions?

1. Wrap Up
   1. Any additional comments/questions?
      Thank participant for participation
   2. Discuss any device return issues
   3. Describe next steps for receiving gift card payment
